# Supplementary material for: Splice-Junction-Based Mapping of Alternative Isoforms in the Human Proteome
Source: Cell Rep. Author manuscript; Available in PMC 2020 Jan 15. (PMC6961840; doi:10.1016/j.celrep.2019.11.026)
Supplement: 2 [file NIHMS1546469-supplement-2.zip › DF1/PXD006675/PulmonaryVein/PulmonaryVein_29_SRSF11_HSITAGPGPSGGPGGGGGGGGGGGGTEVIQVTNVSPSASSEQMR.pdf]

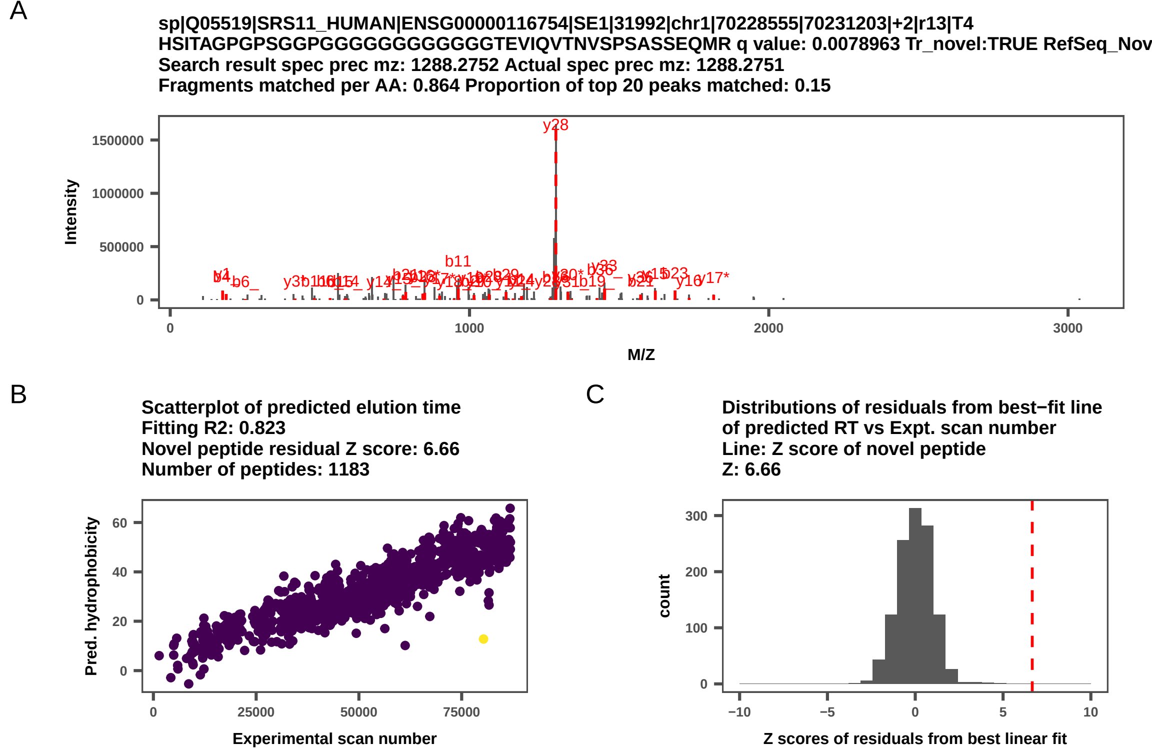
A
sp|Q05519|SRS11_HUMAN|ENSG00000116754|SE1|31992|chr1|70228555|70231203|+2|r13|T4
HSITAGPGPSGGPGGGGGGGGGGGTEVIQVTNVSPSASSEQMR q value: 0.0078963 Tr_novel:TRUE RefSeq_Nov
Search result spec prec mz: 1288.2752 Actual spec prec mz: 1288.2751
Fragments matched per AA: 0.864 Proportion of top 20 peaks matched: 0.15
Intensity
1500000
1000000
500000
0
y28
b11
0
1000
2000
3000
M/Z
B
Scatterplot of predicted elution time
Fitting R2: 0.823
Novel peptide residual Z score: 6.66
Number of peptides: 1183
Pred. hydrophobicity
60
40
20
0
0
25000
50000
75000
Experimental scan number
C
Distributions of residuals from best−fit line
of predicted RT vs Expt. scan number
Line: Z score of novel peptide
Z: 6.66
count
300
200
100
0
−10
−5
0
5
10
Z scores of residuals from best linear fit
